# Stranded because of exhaustion while high-altitude mountaineering in the Swiss Alps: a retrospective nationwide study

**DOI:** 10.1038/s41598-022-12917-8

**Published:** 2022-05-30

**Authors:** Benedikt Gasser

**Affiliations:** Department for Sport, Exercise and Health, University of Basel, Dr. Benedikt Andreas Gasser, Mittlere Allee 18, 4052 Basel, Switzerland

**Keywords:** Disease prevention, Health care, Risk factors

## Abstract

Fortunately, fatal accidents while high-altitude mountaineering have decreased in recent years, but the number of emergencies has increased. These nonfatal emergencies might represent situations where alpinists are stranded (emergencies in which alpinists are no longer able to continue their tour on their own because of, for example, exhaustion, equipment problems, or weather). We analyzed 4596 cases of high-altitude-mountaineering emergencies in the period 2009 to 2020 from the SAC (Swiss Alpine Club) emergency registry. In total, 1951 cases (41.6%) were due to being stranded, 1348 cases were due to falls (28.7%), and 352 cases were due to illness (7.5%); these were the three most common classes. In cases of being stranded, 90% of alpinists were uninjured or not seriously injured. In addition, we found only eight fatal cases. More than 50% of cases occurred while ascending a summit above 4000 m. The main causes of becoming stranded were exhaustion and weather changes. These findings suggest that alpinists contact rescue organizations before experiencing serious troubles; these situations thus present risks and dangers both to those stranded and to emergency services. Since exhaustion and weather changes are the two main causes, adequate preparation and tour planning may help prevent such emergencies.

## Introduction

During the summer, news tickers often report mountain emergencies^1^. The stories are often similar: three alpinists want to climb the Matterhorn but only reach the summit shortly before sunset^1^. Because of the approaching darkness, descent is no longer possible, so they have to be evacuated by emergency services^1^. If the weather is bad, evacuation might only be possible in the early hours of the following morning, and then the alpinists return, having spent the night at 4400 m in the cold, with slight hypothermia^1^. This is quite typical for a mountain emergency in the Swiss Alps and illustrates the dangers posed by high-altitude mountaineering. It suggests that using emergency services may sometimes be necessary even if alpinists carefully study the weather forecast and plan their tour. Considering these (sometimes unavoidable) dangers, it may be surprising that while the number of members of the German Alpine Association has steadily increased since the 1950s, the rate of fatal accidents has decreased^2^. But while the rate of fatalities has gone down, the number of mountain rescue operations have increased since the 1990s, in both absolute and relative terms. The largest portion of rescues were in response to situations where alpinists were stranded^2^. This was also true for fatal mountain emergencies in the Swiss Alps. From 1984 to 2020, 122 fatal accidents occurred on average per year in the Swiss Alps (Supplemental Fig. 1)^3^. There was an average of 33 deathly accidents per year while high-altitude mountaineering, and the number of fatalities while high-altitude mountaineering decreased over the observation period (2009–2020)^3^. In contrast, the total number of emergencies while high-altitude mountaineering increased^3^. Is the increase in emergencies a consequence of alpinists being stranded? Being stranded encompasses mountain emergencies where climbers are no longer able to continue their tour on their own due, for example, to exhaustion, equipment problems, or weather^4,5^. It can be assumed that bad weather, weather changes, or high altitude can have an impact^6–8^. In addition to other objective (rockslides, fresh snow, winds, etc.) and subjective (overestimation, group dynamics) hazards, the reduced partial pressure of oxygen at high altitudes places higher requirements on the cardiopulmonary system^6–8^. While all these dangers exist, one is tempted to assume that such events should be less likely to occur due to recent technological advancements, such as GPS or altimeters, which make it easier to locate one’s current position to call for help^9,10^. Weather forecasts have also constantly improved, making more adequate tour planning possible and reducing the likelihood of having to call emergency services. But while it may seem that mountain emergencies should be less likely, at least in relative terms, a rough analysis suggests that cases have increased. There has not yet been a detailed analysis of the different causes and consequences of being stranded while high-altitude mountaineering in the Swiss Alps. We hypothesize, however, that the number of cases of stranded high-altitude mountaineers has not changed in recent years and that the severity of the emergencies has not changed either^11^.

## Methods

### Analyzed population

For the analysis, all cases of high-altitude-mountaineering emergencies in the SAC central registry from the period 2009–2020 were considered. The central registry contains data from the Swiss Air Rescue Service (REGA), Air Glaciers Lauterbrunnen, Air Glaciers Saanenland, the Register SAC, the KWRO (Kantonale Walliser Rettungsorganisation), the Snow and Avalanche Research Institute Davos, and the cantonal police registers. The term *mountain emergency* covers any event where mountaineers ask for help from mountain rescue services or are affected by subjective or objective mountain hazards^6–8^. This includes illnesses and evacuations of uninjured mountaineers. Being stranded (or blocked) is a mountain emergency where alpinists can no longer continue their tour on their own due, for example, to exhaustion, equipment problems, or weather^4,5^. Each mountain emergency in the registry included an emergency number, the date, rescue organization, event, place, canton, activity, a NACA score (National Advisory Committee for Aeronautics score), the nationality of the victim, their birth date, sex, place of residence, the coordinates, and a short report^12,13^. We analyzed all cases of being stranded in detail. Ethical approval for secondary data analysis was obtained from the Ethics Committee of Northwestern and Central Switzerland (EKNZ).

### Data preparation

In the first step, we classified the causes of mountain emergencies. In the 12-year period from 1 January 2009 to 31 December 2020, the central registry encompassed a total of 4687 (1027 female and 3660 male) cases where mountain rescue services responded to high-altitude mountaineers in the Swiss Alps. Among those cases, we found 1952 cases of being stranded. These cases were analyzed in detail with regard to age, sex, the time of occurrence, the severity of the event based on the NACA score, and the information from the case reports^14^.

### Statistical analyses

We calculated the mean and standard deviation of the NACA scores for the main classes of cases (stranded, falls, illnesses, etc.) for the entire sample. To analyze differences in the severity of the emergencies between the different causes, we performed an ANOVA with post hoc Ganges–Howell tests. For the cases of being stranded, we calculated descriptive statistics for age and NACA scores for women and men. To analyze potential differences in age and NACA scores for cases of being stranded, Jarque–Bera tests^15^ were performed to determine if there was a normal distribution, and Mann–Whitney U tests had to be performed because there was not a normal distribution. To analyze changes in the number of cases and NACA scores over the observation period, linear regressions with calculations of the coefficient of determination (*R*^2^) were performed. Calculations were made with Microsoft Excel (Microsoft Inc., Redmond, WA, USA) and SPSS (Armonk, New York, USA).

## Results

**Figure 1:**
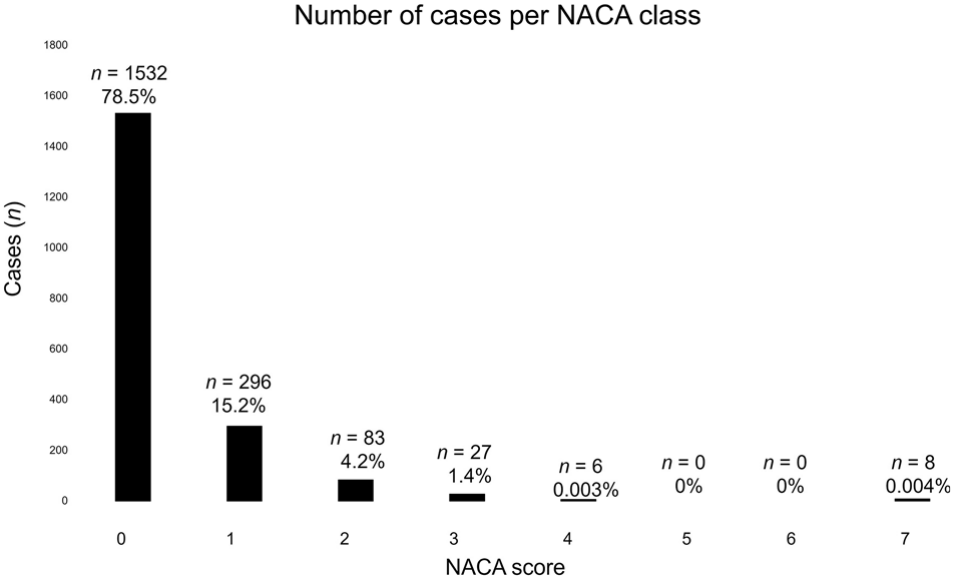
Most victims were uninjured, and only eight fatal cases could be identified. This is further evidenced by the average NACA score for the total sample of 0.32 ± 0.71.

**Figure 2:**
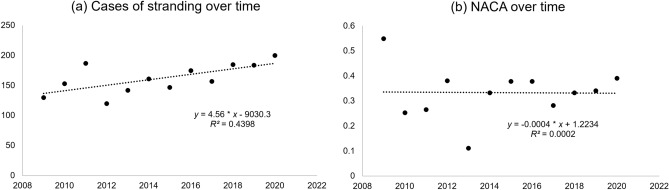
(**a**) Cases of being stranded over the observation period (2009–2020) based on the regression model (n = 4.5629 * time + 132.09) with a high degree of variance detection of R^2^ = 0.4398. A total increase from 137 to 187 cases of approximately 36% of cases is estimated, yielding an average increase per year of 2.6%. (**b**) NACA scores of cases of being stranded over the observation period (2009–2020).

In the total of 4687 (1027 female and 3660 male) emergencies, the most common cause was being stranded with 1951 cases (41.6%), followed by falls with 1348 cases (28.7%), illnesses with 352 cases (7.5%), and being lost with 275 (5.8%) cases (Table 1). Being stranded is thus by far the most common reason for receiving help from emergency services. Of the 1951 cases of being stranded, 1557 were male alpinists and 394 were female alpinists. The cases of being stranded had a low average NACA score of 0.32 ± 0.71; 1532 cases (78.5%) had a NACA score of 0; 296 cases had a NACA score of 1 (15.2%); 83 cases (4.2%) had a NACA score of 2; 27 cases (1.3%) had a NACA score of 3; 6 cases (0.3%) had a NACA score of 4; and 8 cases (0.004%) had a NACA score of 7; the eight cases with a NACA score of 7 were fatal (Fig. 1). Our analysis of changes in the NACA scores over the observation period suggests there was little change in the severity of the injuries (*R*^2^ = 0.0002) (Fig. 2b).

**Table 1:** Overview of mountain emergencies.

Class	Number of cases	Percent of total sample	NACA score Mean ± *SD*
Stranded	1951	41.6	0.3 ± 0.7
Fall	1348	28.7	3.5 ± 2.2
Illness	352	7.5	2.3 ± 1.4
Lost	275	5.9	0.3 ± 0.6
Rockslide	266	5.7	2.8 ± 1.8
Crevasse	162	3.5	1.9 ± 1.6
Avalanche	45	1.0	2.3 ± 2.2
Other	292	6.2	1.6 ± 1.9
Total	4691	100%	1.7 ± 1.8

“Other” included causes such as lightning strikes, equipment failures, and cases where no cause was listed. There was a statistically significant difference in NACA scores (F(8, 4753) = 578.1, p < 0.01). The Ganges–Howell test for multiple comparisons revealed a significant difference between being stranded and all the other classes except for being lost, which implies that the injuries in emergencies due to being stranded or being lost are less severe than those of the other classes, such as falls, illnesses, rockslides, crevasses, and avalanches.

The average age of females was 41.3 ± 11.3 years, and the average age of males was 42.8 ± 13.1 years, which was not significantly different (*p* = 0.186). The average NACA score was 0.334 ± 0.62 for females and 0.319 ± 0.74 for males (*p* = 0.415).

In the few cases where there was an injury, it concerned one of the following: 8 cases of leg injuries (6.5%), 33 cases of hand injuries (26.8%), 10 cases of shoulder injuries (8.1%), 9 cases of knee injuries (7.3%), 13 cases of head injuries (10.6%), 12 cases of foot injuries (9.8%), 22 cases of hypothermia (17.9%), and 16 cases of frostbite (13.0%).

In the observation period, an average of 161.8 ± 24.8 cases of being stranded were detected per year. Based on our regression model, such cases increased over the observation period (*n* = 4.5629 * time + 132.09) with a high degree of variance, *R*^2^ = 0.4398 (Fig. 2a).

**Figure 3:**
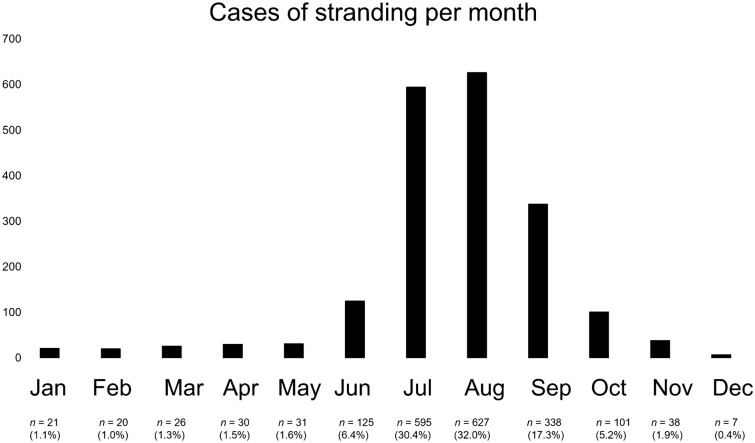
Most cases of being stranded were in the summer months of July and August.

Looking at the time of occurrence, cases were mainly in the two summer months of July and August (Fig. 3).

A significant cause of being stranded was weather. In 76 cases (12.7%), thunderstorms were identified as the cause, fog was identified in 73 cases (12.2%), weather changes in 59 cases (9.8%), and fresh snow in 34 cases (5.7%). However, the most common reason for being stranded was exhaustion, with 357 cases (59.6%).

**Figure 4:**
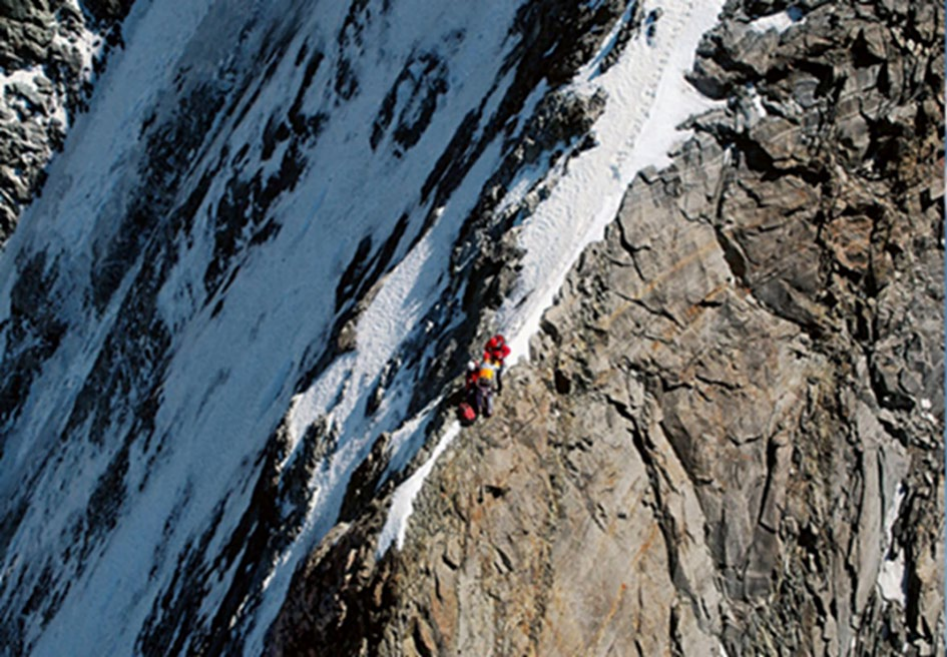
In almost 80 percent of the analyzed cases, alpinists were not injured when stranded, often on a classic route on a peak over 4000 m. A typical situation of being stranded is shown on the left on the Zmuttgrat on the Matterhorn. Alpinists overestimated their abilities and did not have enough time to return. A helicopter with longlines is then often the only possibility for rescue.

A very high share of the cases were on popular mountain tours. Nearly one-third of the cases (551 cases, 28.1%) were close to a summit over 4000 m, and a total of 1073 (54.9%) cases were on a route to a peak over 4000 m. There were 223 cases (11.4%) on the Matterhorn (Fig. 4) and 141 cases (7.2%) on the Piz Bernina.

Concerning the nationality of the victims, 668 cases (34.2%) were from Switzerland, 426 cases (21.8%) were from Germany, 120 cases (6.1%) were from Italy, 110 cases (5.6%) were from France, and 85 cases (4.3%) were from Austria. So approximately 70 percent were mountaineers from countries where the Alps are located. In addition, there were 81 cases from the Czech Republic (4.1%), 64 from Poland (3.2%), 64 from Great Britain (3.2%), 54 from Spain (2.8%), 40 from the Netherlands (2%), 39 from Belgium (2%), 19 from Romania (1%), and 8 or fewer from each of Japan, Finland, and Canada.

## Discussion

The aim of this study was to analyze mountain emergencies due to being stranded in recent years in the Swiss Alps. Based on the increasing number of cases per year, one might be tempted to assume that cases of being stranded have increased. The number of cases increased, however, by only approximately 2.6% per year, which is lower than the increase in SAC members of approximately 4% per year during the same time period. This number may be used as a proxy for activity in the mountains, so it suggests that there has not been an increase in the rate of cases of being stranded but rather, if anything, a decrease. Based on their NACA scores, we found no change in the severity of the emergencies over time. The most frequently mentioned cause of being stranded in the case reports was exhaustion, which is in line with findings of high physical requirements of high altitude-mountaineering^16–19^. In addition to exhaustion, factors associated with weather, such as thunderstorms, fog, and fresh snow, were identified as reasons for contacting emergency services. The fact that most of the cases occurred in the summer months is to be expected, since most routes on peaks over 4000 m are best undertaken in the sunniest periods of the year^20^. In summary, mountaineering has become increasingly popular^4,21–23^, and being stranded is the most common cause of contacting emergency services, accounting for 41.6% of cases. The majority of alpinists in cases of being stranded were not injured, and only 8 fatal cases were found in the whole observation period. Interestingly, a very high number of cases occurred on two very popular mountains, the Matterhorn and the Piz Bernina. These two mountains accounted for almost 20% of all the cases of being stranded. It seems likely that on these two popular peaks, alpinists often want to reach the peak but are not trained well enough. This results in becoming stranded due to exhaustion. As indicated in Fig. 4, cases of stranded alpinists are often not only challenging for victims but also for emergency services.

Regarding limitations, the study design must be mentioned. The study design consisted in a retrospective analysis, but a prospective design would have been superior at identifying potential causes of emergencies in greater detail. Directly after an accident, one can speak with victims and rescue services, which results in a more precise reconstruction of the situation than one can obtain from analzying a short ten-year-old case report. This would also provide a direct empirical basis for preventative measures, which, for example, the SAC could then implement.

To conclude, being stranded is the most common cause of emergencies in high-altitude mountaineering in the Swiss Alps. Normally, victims are not severely injured, so the greatest consequence of being stranded is the burden and costs of emergency services. Since a majority of the cases seem to be caused by exhaustion or weather factors, such emergencies might be preventable. Training appropriately and good tour planning with careful consultation of the weather forecasts can prevent situations where alpinists have to contact emergency services. Prevention measures should focus on promoting adequate training and planning before one undertakes a tour in Switzerland’s highest mountains.

## Supplementary Information

Supplementary Legends.

Supplementary Figure 1.

## Data Availability

The datasets generated and/or analysed during the current study are not publicly available due to protection of personal rights but are available from the corresponding author on reasonable request.
