# Supplementary material for: Stranded because of exhaustion while high-altitude mountaineering in the Swiss Alps: a retrospective nationwide study
Source: Sci Rep. 2022 May 30;12:9011. doi: 10.1038/s41598-022-12917-8 (PMC9151813; doi:10.1038/s41598-022-12917-8)
Supplement: Supplementary file 1 — Supplementary Legends. [file 41598_2022_12917_MOESM1_ESM.docx]

**Supplemental Figure 1** *Fatal mountain emergencies in the Swiss Alps from 1984 to 2020. The number of fatal emergencies decreased despite the fact that the number of alpinists strongly increased over the observation period, as suggested by the 4% annual increase in the number of members of the SAC^19^.*
