# Supplementary figures and images for: Stranded because of exhaustion while high-altitude mountaineering in the Swiss Alps: a retrospective nationwide study
Source: Sci Rep. 2022 May 30;12:9011. doi: 10.1038/s41598-022-12917-8 (PMC9151813; doi:10.1038/s41598-022-12917-8)

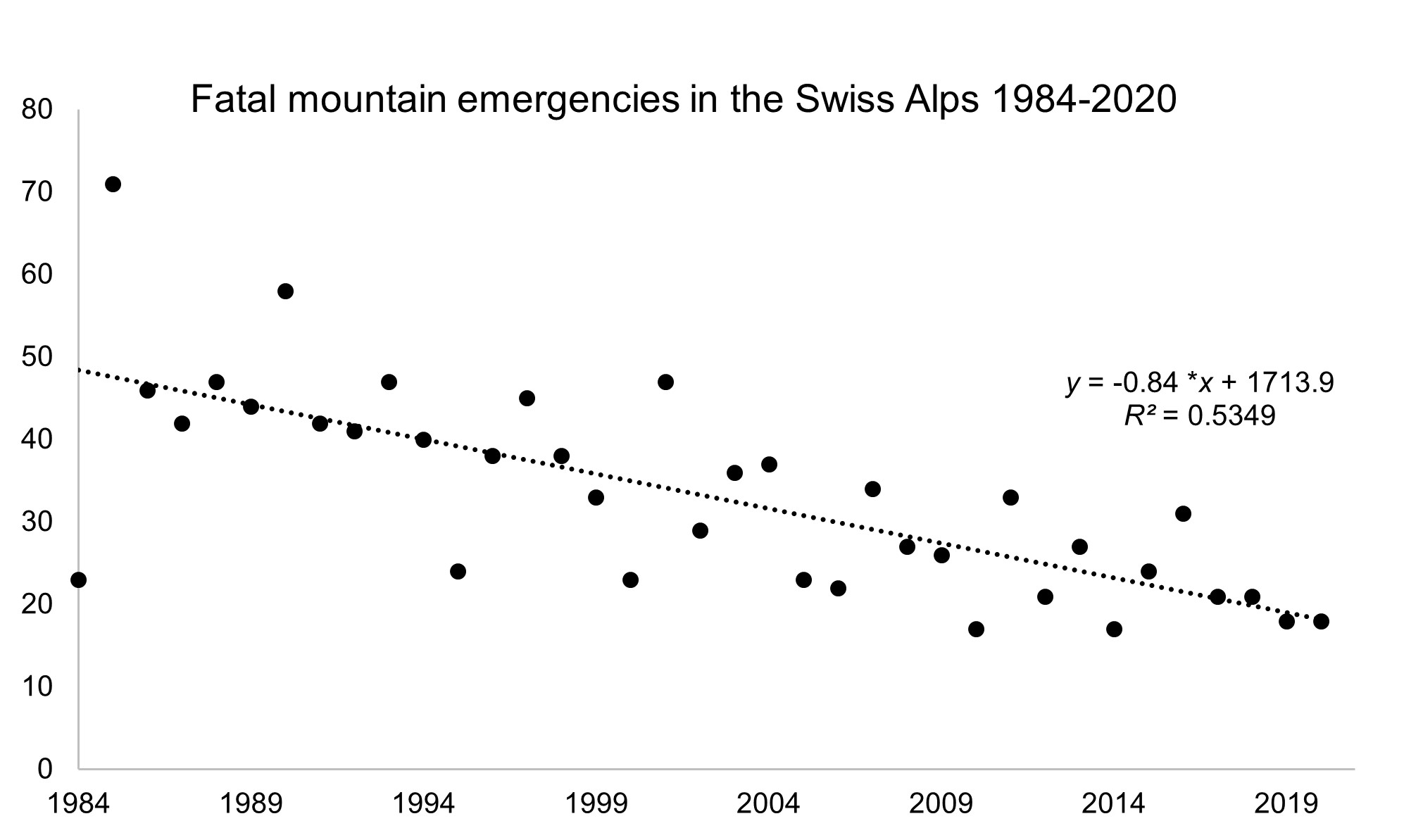

Supplement: Supplementary file 2 — Supplementary Figure 1. [file 41598_2022_12917_MOESM2_ESM.jpg]
